# Blepharoplasty: An Overview

**DOI:** 10.4103/0974-2077.53092

**Published:** 2009

**Authors:** Milind N Naik, Santosh G Honavar, Sima Das, Savari Desai, Niteen Dhepe

**Affiliations:** 1 *Department of Ophthalmic and Facial Plastic Surgery, The Aesthetics Clinic, LV Prasad Eye Institute, Hyderabad, Andhra Pradesh, India*; 2 *Skin City Postgraduate Dermatology Institute, Pune, Maharashtra, India*

**Keywords:** Blepharoplasty, fat excision, fat repositioning

## Abstract

Blepharoplasty plays a vital role in facial rejuvenation, with direct aesthetic relation to the brow and the cheek. Upper and lower eyelid blepharoplasty are indicated for the treatment of excess skin and/or orbital fat. Preoperative evaluation should include a thorough medical and ophthalmic history, along with a detailed cutaneous and eye examination. Symptoms of preexisting dry eye should be elicited preoperatively, as they directly correlate with postoperative complications. Physical examination should take into account brow position, eyelid ptosis, lower eyelid position, and cheek projection. Blepharoplasty can be performed by many operative approaches. This review highlights the standard skin-only upper blepharoplasty and lower eyelid conservative fat excision or repositioning.

## INTRODUCTION

Ophthalmic plastic surgery primarily deals with disorders of the eyelid, lacrimal apparatus, orbit and periocular cosmetic surgery. In the past, the average ophthalmic plastic surgeon focused mainly on the functional disciplines, with few surgeons showing any interest in cosmetic surgery. This trend is gradually changing, and today, most ophthalmic plastic surgeons perform cosmetic surgeries and many specialize in this area of practice.

The eye is an important component of facial aesthetics, and blepharoplasty can play a vital positive role in facial harmony and the perception of aging. Blepharoplasty is one of the most commonly performed facial cosmetic procedures. Symptoms such as tired-looking eyes, excess skin, droopy eyelids, or circles around the eyes may benefit from blepharoplasty.[1] It can also be combined with other facial and skin rejuvenation procedures such as brow or mid-face lift, lasers or chemical skin resurfacing.[2] This article aims to give an overview of upper and lower eyelid blepharoplasty techniques.

## PREOPERATIVE EVALUATION

Preoperative patient evaluation for blepharoplasty should document medical and ophthalmologic history such as chronic systemic diseases and medications. Ophthalmologic history should be obtained, including vision, corrective lenses, trauma, glaucoma, allergic reactions, excess tearing, and dry eyes. No cosmetic surgery of the periorbital region should be performed for a minimum of six months following corneal refractory surgery. Schirmer's test should be considered if there is history of dry eye.

In addition to complete eye examination, the evaluation of the periorbital area should take into account skin quality and quantity, underlying three-dimensional soft-tissue contours, and the bony skeletal support.

### Assessment of the upper eyelid

**Figure 1:**
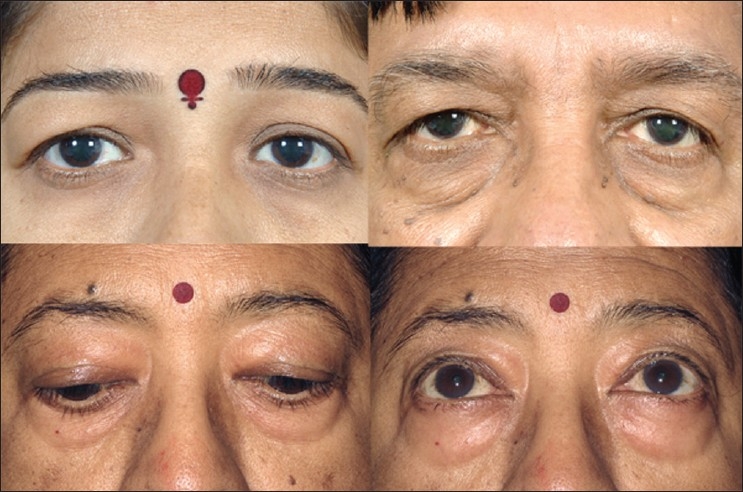
Typical aging changes in the eyelid. Overhanging upper eyelid skin (top left and right), with prominent orbital rim hollow and lower eyelid fat prolapse (top right). Lower eyelid fat prolapse becomes less prominent in downgaze (bottom left) and more prominent in upgaze (bottom right)

Upper eyelid dermatochalasis is the loss of elasticity and support in the skin. This can create a fold of excess upper eyelid skin, which can impair the function of the eye, including supero-lateral visual field obstruction [Figure 1, top left and right]. Evaluation of pre-septal and eyebrow fat pads is important in redefining the superior sulcus. Assessment of patient's old photographs aids the surgeon in restoring the youthful look. Upper eyelid ptosis should also be noted, since it can be corrected simultaneously.

### Assessment of the lower eyelid

Lower eyelids should be assessed for skin excess and fat herniation, which typically presents as medial, central, and lateral fat pads. Lower eyelid fat becomes more prominent in upgaze and less prominent in downgaze [Figure 1, bottom left and right]. Downward displacement of the lateral canthus can result from disinsertion, laxity, or the presence of a prominent eye [Figure 1, top right]. Lower lid distraction test can determine the degree of laxity and guide lower eyelid canthal repositioning. The posterior displacement of the orbital rim in relation to the anterior cornea and lower lid margin, a negative vector, should be noted preoperatively. Prominent or deep-set eyes should be documented with exophthalmometry. Malar anatomy needs to be evaluated for periorbital hollows.

### Assessment of the eyebrow

Brow ptosis is assessed by evaluating the position of the eyebrow in relation to the superior orbital rim. Asymmetry in the upper and lower eyelids and brow position is common and should be recognized and addressed individually.

## ANESTHESIA

Blepharoplasty may be performed under either local or general anesthesia depending upon the surgical plan, patient and surgeon preference, and need for concomitant operations. A simple upper or lower eyelid blepharoplasty where only skin or fat is excised can be performed under local anesthesia. Other more invasive procedures, such as lower blepharoplasty combined with fat repositioning, mid-face lift, or endoscopic browlift may need intravenous sedation, or general anesthesia.

## UPPER EYELID BLEPHAROPLASTY

### Preoperative marking

**Figure 2:**
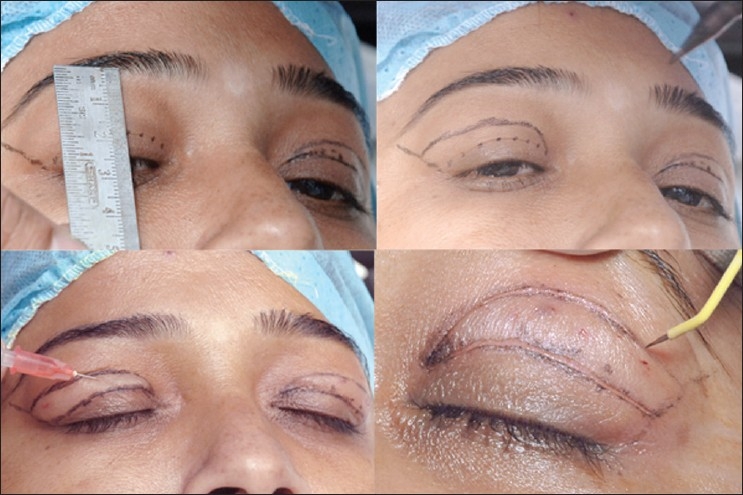
Upper eyelid blepharoplasty. Measurement is performed to leave minimum 20 mm vertical lid height (top left). Skin marking for upper eyelid blepharoplasty (top right). Subcutaneous injection of local anesthetic agent (bottom left). Skin incision with radiofrequency fine monopolar empire tip (bottom right)

Preoperative markings should be made with the patient sitting upright in neutral gaze with the brow properly positioned. The eyelid crease is situated above the ciliary margin approximately 8 to 9 mm in women and 7 to 8 mm in men. The lower limit of excision should be along the eyelid crease, and the lateral extent of the marking should be limited by an imaginary line joining the lateral end of the brow to the lateral canthus [Figure 2, top right]. The extent of excision should be at least 10 mm from the inferior border of the brow, making a pattern of skin excision as shown in the Figure 2. A skin pinch test can confirm the preoperative markings. A minimum of 20mm of vertical lid height should be preserved for normal eye closure. The location of fat should be determined and marked preoperatively.

### Surgical technique

**Figure 3:**
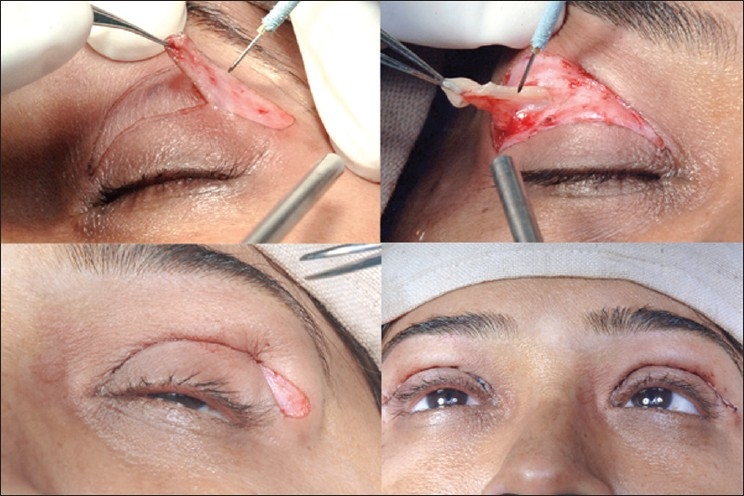
Skin undermining and excision with radiofrequency monopolar tip (top left). Fat pad excision after incising the orbital septum (top right). Periosteal anchoring suture which helps in restoring brow fat pad fullness (bottom left). Skin closure with 6-0 Prolene™ continuous suture (bottom right)

The upper lids should be injected superficially, with 2% lidocaine with 1:100,000 epinephrine using a 27 to 30-gauge needle [Figure 2, bottom left]. Skin incision can be made either with a No 15 Bard Parker blade or the Empire tip of radiofrequency monopolar cautery [Figure 2, bottom right]. Conservative fat excision can be performed as part of upper lid blepharoplasty [Figure 3, top right]. There are two fat compartments, medial and central that can be accessed through small incisions in the septum, teased out, and resected using radiofrequency monopolar tip. Of the medial and central fat, only the fat that comes easily into the wound is excised. It is important not to aggressively pull fat from the orbit.

**Figure 4:**
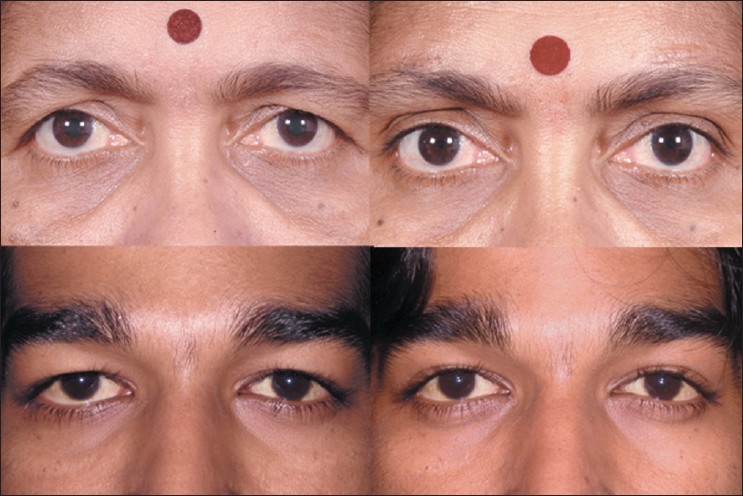
Pre- and postoperative photographs following upper eyelid blepharoplasty combined with external brow-lift (top left and right); and upper eyelid blepharoplasty combined with retro-orbicularis oculi fat (ROOF) excision (bottom left and right)

Retro-orbicularis oculi fat can be accessed beneath the lateral orbicularis overlying the superior orbital rim. Resection has been described to help decrease heaviness of the upper lid and lateral brow. The sub-brow fat pad can be repositioned during wound closure with use of eyelid suspension sutures. This can be done with two to three absorbable sutures that incorporate the orbicularis from the lower and upper edge of the incision along with the superolateral arcus marginalis [Figure 3, bottom left]. These sutures might result in early over-correction of the upper eyelid leading to lagophthalmos, which improves within days after the surgery. The transconjunctival approach has very limited application in upper eyelid blepharoplasty.[3] The skin incision can be closed using running or interrupted sutures with various absorbable or permanent materials [Figure 3, bottom right], or cyanoacrylate glue to achieve an aesthetic outcome [Figure 4].

### Postoperative care

Postoperatively, patients should be advised to use ice packs on the surgical site for three days to minimize postoperative swelling, and topical ciprofloxacin ophthalmic ointment on the incision sites for two weeks. Non-absorbable sutures, if used, can be removed after one week.

### Complications

Possible complications include upper eyelid retraction with scleral show from anterior lamellar inadequacy, lagophthalmos, acquired diplopia and corneal exposure. The most common complication of cosmetic surgery is failure to meet the patient's expectations. This can be avoided by preoperative counseling and identifying reasonable expectations.

## LOWER EYELID BLEPHAROPLASTY

Aging can lead to a number of aesthetic changes in the lower eyelid including skin laxity or excess, orbital septum laxity, orbicularis laxity or hypertrophy, herniation of the orbital fat, canthal laxity, malar festoons, crow's feet, and periocular wrinkles.[4] Common complaints include eyelid bags, circles under the eye, wrinkles around the eye, or a tired look. Anatomically, relaxation of the orbital septum, orbicularis muscle, and skin can cause protrusion of intraorbital fat leading to eyelid bags [Figure 1, top right]. Typical periorbital hollows have a distinct anatomic basis that needs attention during treatment.[5] Lower eyelid rejuvenation is more complex and has several treatment options such as fillers, blepharoplasty, and skin resurfacing. Most cosmetic surgeons today have developed a customized approach to eyelid surgery in which the specific anatomic problems are identified and the operation is individualized to address these problems.

The traditional procedure in lower eyelid blepharoplasty was to remove the pseudo-herniated fat via skin incision. A recent, more conservative approach includes repositioning of the herniated fat in cases of tear trough deformity into the subperiosteal space. Both these approaches may be accompanied by strengthening procedures for the attenuated septum or septorrhaphy and tightening of the orbicularis muscle and skin.[6] Defocused CO_2_ laser irradiation of the undersurface of the orbicularis results in persistent shortening and tightening of the muscle tissue.[4]

The approach to the lower eyelid remains a controversial issue within plastic surgery. The transconjunctival approach had reduced chances of eyelid retraction, scleral show, and postoperative ectropion compared to other methods.[7–9] Some surgeons prefer a transcutaneous approach in patients who have hypertrophy of the orbicularis oculi muscle and therefore require muscle excision.[10]

### Transconjunctival lower lid blepharoplasty

**Figure 5:**
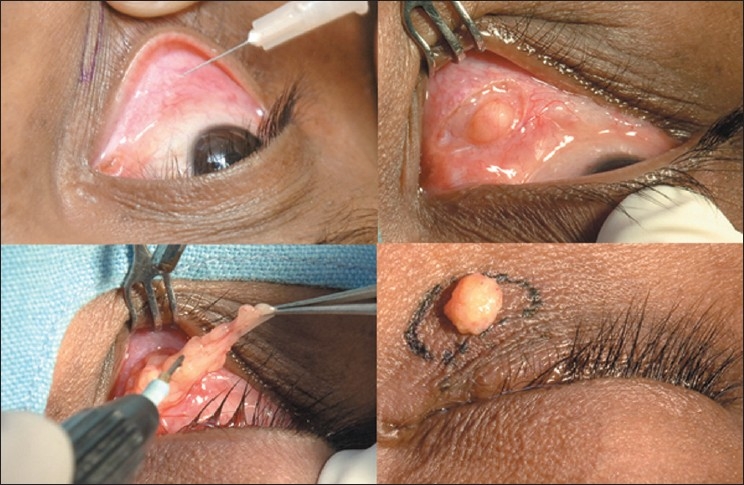
Transconjunctival blepharoplasty (surgeon's view). Injection of local anesthetic into the inferior conjunctival fornix (top left). Forniceal incision to expose orbital fat (top right). Conservative fat excision with radiofrequency monopolar tip (bottom left). Assessment of contours on table (bottom right)

**Figure 6:**
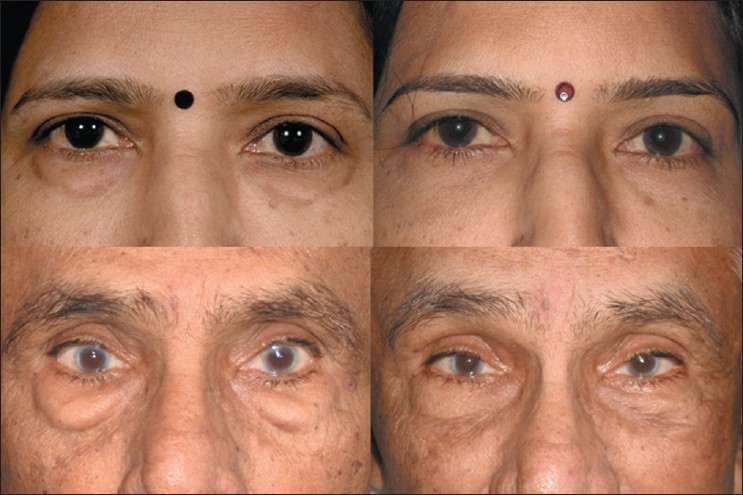
Pre-and postoperative photographs following transconjunctival lower eyelid blepharoplasty involving fat excision only (top left and right); and transconjunctival lower eyelid fat repositioning (bottom left and right)

For transconjunctival lower lid blepharoplasty, the inferior fornix, eyelid skin, and lateral canthus are anesthetized with 1% lidocaine containing 1:100,000 epinephrine [Figure 5, top left]. Surgery is performed through an incision made 4-6 mm below the lid margin through the conjunctiva and lower eyelid retractors [Figure 5, top right]. This can be done by sharp dissection with scissors, radiofrequency monopolar cautery, or laser. Gentle pressure on the eyeball prolapses the fat compartments and aids in identification of the medial, central, and lateral fat pads. Conservative fat removal is achieved using radiofrequency monopolar or bipolar cautery [Figure 5, bottom left and right]. Care is taken not to damage the inferior oblique muscle that separates the medial from the central fat pocket. The endpoint for excision is reached when gentle pressure on the globe results in the anterior aspect of the orbital fat being flush with the orbital rim. The tarsoconjunctival incision is left unclosed, but the inferior and superior edges of the conjunctival epithelium are well apposed to avoid overlap. The incision usually heals within a week to give an aesthetic result [Figure 6, top left and right].

Some surgeons perform lateral canthopexy along with lower eyelid blepharoplasty. It eliminates unnecessary skin resection, and is believed to restore tone and youthful contour of the lower eyelid.[4,11,12]

Wrinkles and excess of vertical skin can be dealt with by skin resurfacing techniques, such as chemical peeling and CO_2_ or Erbium-YAG laser resurfacing. These techniques are applicable only to patients with Fitzpatrick skin Type I-III. Patients with darker skin are at risk of pigmentary changes; hence these can be managed with surgical excision by the pinch technique (see below).

### Transcutaneous lower lid blepharoplasty

The ‘skin pinch’ technique is ideal for skin laxity alone, with no fat prolapse.[13] It is approached through a subciliary incision with the skin elevated off the orbicularis. The amount of skin to be resected can be estimated with a skin pinch between forceps. Redundant skin can be removed conservatively and redraped without disturbing the underlying orbicularis.

The more aggressive ‘skin-muscle flap’ method is also approached through a subciliary incision, undermining the skin and orbicularis. The pretarsal orbicularis fibers should remain intact, and the skin and preseptal orbicularis are elevated as one flap. Dissection can be continued along the orbital septum to the level of the orbital rim. Periorbital fat is approached through small incisions in the septum. Orbicularis muscle fibers and skin can be excised at closure; however, damage to the orbicularis may lead to lower lid malposition and orbicularis denervation.

### Complications

Severe complications, such as visual loss from orbital hemorrhage, orbital injection, or posterior optic nerve infarction are extremely rare, but have been described.[14] Other possible complications are lower eyelid retraction with scleral show, lagophthalmos, corneal exposure and acquired strabismus.

### Lower eyelid fat repositioning

In youth, the eyelid-cheek complex has a single smooth convex profile. With advancing age, thinning of septum, receding bony orbital rim, and pseudo-herniation of intraorbital fat occurs producing a double convex deformity on the lower eyelid.[15,16] Simple removal of orbital fat in this situation can result in a hollow appearance of the lower eyelid. Preservation of the lower orbital fat is a new concept in facial rejuvenation, and such preservation creates a smooth transition to the malar eminence [Figure 6, bottom left and right], blending nicely into the upper face.[15,17]

### Surgical technique

**Figure 7:**
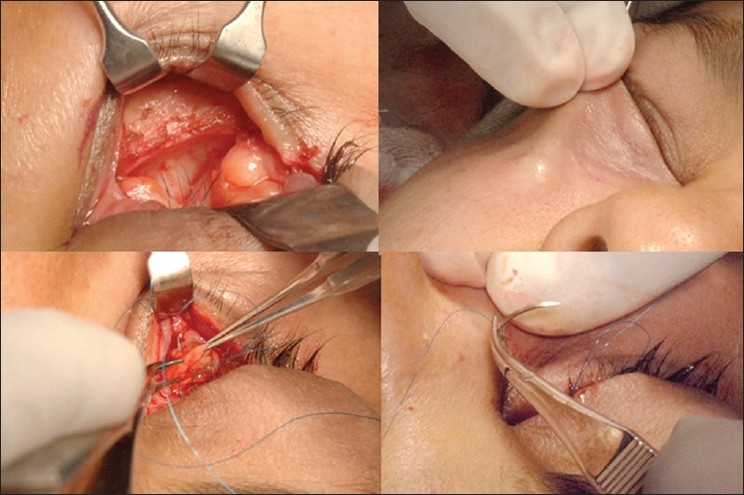
Transconjunctival lower eyelid fat repositioning. Surgeon's view showing exposed orbital fat and inferior orbital rim (top left). Release of orbitomalar ligament and creation of supra-periosteal pocket for fat repositioning (top right). Prolene^™^ 6-0 sutures passed through orbital fat pads (bottom left). Exteriorization of Prolene™ sutures to reposit fat within the orbital rim hollow (bottom right)

Fat repositioning surgery includes release of the arcus marginalis and advancement of the orbital fat beyond the infraorbital rim underneath the orbicularis muscle with the help of temporary exteriorized sutures [Figure 7]. The fat can be placed either in the subperiosteal or supra-periosteal plane, with no apparent effect on aesthetic results. This technique camouflages the lower orbital rim anatomy and provides more youthful rejuvenation of the mid-face.[15,18,19]

Other methods suggested to correct tear trough deformity include orbital fat removal, fat injections or grafts, and alloplastic cheek implants. In general, transconjunctival fat repositioning results in leveling of the tear trough deformity, a smooth contour of the lower eyelid, and high patient satisfaction.[20]

### Complications

Complications include persistence of dark circles, temporary skin irregularities from fat and edema, fat granulomas, restricted ocular motility, or new-onset diplopia.[21]

## COMBINATION THERAPY

Variations to these standard blepharoplasty approaches are numerous, and often a combination therapy is needed based on patient findings.[22] Fillers, such as hyaluronic acid, can be used especially in the lower eyelid to correct volume deficiency that could not be addressed by blepharoplasty alone. Autologous fat transfer into this area is also an option, with long-lasting results. Orbicularis repositioning can be used to eliminate hypotonic and herniated orbicularis muscle, soften palpebral depressions, and shorten the lower lid to cheek distance. Lower eyelid skin resurfacing can be performed with chemical peels or ablative laser for conservative lid tightening as an adjunct to a transconjunctival fat excision. Lateral canthal resuspension is often combined with lower eyelid blepharoplasty as described earlier. Mid-face cheek and orbicularis repositioning, and subperiosteal mid-face-lifting procedures are often combined, but are beyond the scope of this article. Concomitant endoscopic brow lift if performed should be done before upper blepharoplasty so that correct brow position is ensured before upper lid skin excision.

## CONCLUSION

This article briefly describes the techniques of standard upper and lower eyelid blepharoplasty. Practically, the rejuvenation of this complex anatomical area requires a combination of therapies including fat excision, repositioning or transfer, simultaneous brow or mid-face lift, and adjunctive treatment for skin resurfacing and periorbital hollows.
